# Supplementary material for: Assessment of the Effectiveness of Obstructive Sleep Apnea Treatment Using Optical Coherence Tomography to Evaluate Retinal Findings
Source: J Clin Med. 2022 Feb 3;11(3):815. doi: 10.3390/jcm11030815 (PMC8837143; doi:10.3390/jcm11030815)
Supplement: Supplementary file 1 [file jcm-11-00815-s001.zip › jcm-1532334-supplementary.pdf]

Supplementary Materials:

Table S1. Evolution of peripapillary RNFL thicknesses in the mild-moderate OSA group (43 eyes evaluated from 23 patients). GLM: General Linear Model; SD: standard deviation; df: degrees of freedom; eta2: partial eta2 (effect size); p: level of statistical significance.

**Variable (n)**	**Mean ± SD**		**Treatment effect (comparison pre/post) F(d.f.); *p*-value (eta^2)**
	**Pre-treatment**	**Post-treatment**	
**RNFL average thickness (µm)**			F(1.38)=1.254; p=0.270
**CPAP (10)**	95.5 ± 11.1	94.8 ± 9.3	(0.032)
**Surgery (19)**	105.7 ± 8.4	104.9 ± 7.2	
**CPAP + surgery (12)**	102.4 ± 4.6	100.3 ± 5.9	
**Total**	102.2 ± 9.1	101.1 ± 8.3	
**RNFL superior quadrant thickness (µm)**			F(1.38)=0.112; p=0.740
**CPAP (10)**	119.7 ± 11.7	116.8 ± 14.3	(0.003)
**Surgery (19)**	131.7 ± 12.5	130.5 ± 15.0	
**CPAP + surgery (12)**	135.3 ± 10	132.8 ± 8.9	
**Total**	129.1 ± 12.6	128.6 ± 14.8	
**RNFL nasal quadrant thickness (µm)**			F(1.38)=1.692; p=0.201
**CPAP (10)**	71.5 ± 19.9	72.2 ± 18.3	(0.043)
**Surgery (19)**	80.7 ± 14.9	79.5 ± 11.6	
**CPAP + surgery (12)**	71.2 ± 13.9	65.2 ± 9.5	
**Total**	75.7 ± 16.3	73.5 ± 14.1	
**RNFL inferior quadrant thickness (µm)**			F(1.38)=0.460; p=0.502
**CPAP (10)**	117.3 ± 20.7	117.4 ± 19.9	(0.012)
**Surgery (19)**	136.8 ± 13.6	135.7 ± 14.8	
**CPAP + surgery (12)**	124.9 ± 12.1	121.1 ± 11.9	
**Total**	128.6 ± 17	127 ± 17.2	
**RNFL temporal quadrant thickness (µm)**			F(1.38)=0.039; p=0.845
**CPAP (10)**	73.6 ± 7.8	74.7 ± 10.1	(0.001)
**Surgery (19)**	73.6 ± 10.3	72.6 ± 9.7	
**CPAP + surgery (12)**	80.6 ± 14.3	79.7 ± 15.3	
**Total**	75.6 ± 11.3	75.2 ± 11.8	

Table S2. Evolution of peripapillary RNFL thicknesses in the severe OSA group (55 eyes evaluated from 29 patients). GLM: General Linear Model; SD: standard deviation; df: degrees of freedom; eta2: partial eta2 (effect size); p: level of statistical significance.

**Variable (n)**	**Means ± SD**		**Treatment effect (comparison pre/post) F(d.f.); *p*-value (eta^2)**
	**Pre-Treatment**	**Post-treatment**	
**RNFL average thickness (µm)**			F(1.52)=4.121; **p=0.047**
**CPAP (22)**	98.9 ± 9.1	100.8 ± 9.6	(0.067)
**Surgery (10)**	97.2 ± 7.3	99.6 ± 5.9	
**CPAP + surgery (23)**	92.9 ± 11.8	93.8 ± 14.1	
**Total**	**96.1 ± 10.3**	**97.7 ± 11.6**	
**RNFL superior quadrant thickness (µm)**			F(1.52)=1.915; p=0.172

**CPAP (22)**	125.1 ± 16.1	126 ± 13.9	(0.036)
**Surgery (10)**	120.6 ± 20.3	125.3 ± 17.7	
**CPAP + surgery (23)**	120.4 ± 19.7	121.4 ± 20.1	
**Total**	122.3 ± 18.2	124 ± 17.2	
**RNFL nasal quadrant thickness (µm)**			F(1.52)=1.173; p=0.284
**CPAP (22)**	77.4 ± 17.3	80.8 ± 21.1	(0.022)
**Surgery (10)**	73.9 ± 16.1	76.9 ± 16	
**CPAP + surgery (23)**	69.1 ± 11.3	69.6 ± 16.6	
**Total**	73.3 ± 15	75.4 ± 18.8	
**RNFL inferior quadrant thickness (µm)**			F(1.52)=0.001; p=0.979
**CPAP (22)**	120.9 ± 13.4	120.1 ± 29.9	(0.000)
**Surgery (10)**	125.9 ± 13.1	125.6 ± 13.4	
**CPAP + surgery (23)**	116.6 ± 19.1	117.6 ± 21.5	
**Total**	120 ± 16.1	120.1 ± 24	
**RNFL temporal quadrant thickness (µm)**			F(1.52)=0.706; p=0.405
**CPAP (22)**	72.4 ± 11.6	71.7 ± 13	(0.013)
**Surgery (10)**	68.2 ± 9.9	70.7 ± 11.5	
**CPAP + surgery (23)**	65.4 ± 14.9	67.1 ± 15.3	
**Total**	68.7 ± 13	69.6 ± 13.7	
